# Prediction of blood pressure changes associated with abdominal pressure changes during robotic laparoscopic low abdominal surgery using deep learning

**DOI:** 10.1371/journal.pone.0269468

**Published:** 2022-06-06

**Authors:** Yang-Hoon Chung, Young-Seob Jeong, Gati Lother Martin, Min Seo Choi, You Jin Kang, Misoon Lee, Ana Cho, Bon Sung Koo, Sung Hwan Cho, Sang Hyun Kim

**Affiliations:** 1 Department of Anesthesiology and Pain Medicine, Soonchunhyang University Bucheon Hospital, Soonchunhyang University College of Medicine, Bucheon, Republic of Korea; 2 Department of Computer Engineering, Chungbuk National University, Cheongju, Republic of Korea; 3 Department of ICT Convergence, Soonchunhyang University, Asan-si, Republic of Korea; 4 Industry-Academy Cooperation Foundation, Soonchunhyang University, Asan-si, Republic of Korea; Henry Ford Hospital Systems & Outcomes Research Consortium, Cleveland Clinic, UNITED STATES

## Abstract

**Background:**

Intraoperative hypertension and blood pressure (BP) fluctuation are known to be associated with negative patient outcomes. During robotic lower abdominal surgery, the patient’s abdominal cavity is filled with CO_2_, and the patient’s head is steeply positioned toward the floor (Trendelenburg position). Pneumoperitoneum and the Trendelenburg position together with physiological alterations during anesthesia, interfere with predicting BP changes. Recently, deep learning using recurrent neural networks (RNN) was shown to be effective in predicting intraoperative BP. A model for predicting BP rise was designed using RNN under special scenarios during robotic laparoscopic surgery and its accuracy was tested.

**Methods:**

Databases that included adult patients (over 19 years old) undergoing low abdominal da Vinci robotic surgery (ovarian cystectomy, hysterectomy, myomectomy, prostatectomy, and salpingo-oophorectomy) at Soonchunhyang University Bucheon Hospital from October 2018 to March 2021 were used. An RNN-based model was designed using Python3 language with the PyTorch packages. The model was trained to predict whether hypertension (20% increase in the mean BP from baseline) would develop within 10 minutes after pneumoperitoneum.

**Results:**

Eight distinct datasets were generated and the predictive power was compared. The macro-average F1 scores of the datasets ranged from 68.18% to 72.33%. It took only 3.472 milliseconds to obtain 39 prediction outputs.

**Conclusions:**

A prediction model using the RNN may predict BP rises during robotic laparoscopic surgery.

## Introduction

Intraoperative hypertension is known to be associated with negative patient outcomes [1, 2]. Although there is insufficient evidence on the acceptable range of intraoperative hypertension and the actual risk to patients [3, 4], increased intraoperative blood pressure (BP) fluctuation was significantly associated with postoperative poor outcomes [5–7]. Accordingly, maintaining BP within a certain range during surgery is one of the major challenges for anesthesiologists [8].

Changes in BP during surgery are caused by many factors such as underlying disease, the concentration of anesthetic agents, and the surgical position. In cases involving lower abdominal organs such as robotic laparoscopic radical prostatectomy (RALRP) and robotic hysterectomy using a da Vinci robot, the abdominal cavity is filled with CO_2_ gas to create a pneumoperitoneum state. The patient’s head is positioned steeply toward the floor (Trendelenburg position) for the operation. Increased abdominal pressure due to pneumoperitoneum compresses large blood vessels such as the inferior vena cava (IVC) and aorta, reduces blood flow to the mesentery and kidneys, and elevates the diaphragm. Strong compression of the IVC may decrease venous return to the heart, and aortic compression may increase systemic vascular resistance, resulting in increased BP and decreased cardiac output. In addition, the Trendelenburg position can increase the venous return to the heart and offset the reduction in cardiac output [9–11]. During surgery, the Trendelenburg position is implemented immediately after the initiation of pneumoperitoneum. Therefore the two events occur without a significant time difference. Under a combination of these various effects, BP changes are very difficult to predict.

Recent advances in technology have led to several attempts to predict cardiac events such as hypotension during surgery via machine learning (ML) approaches based on various surgical factors with proven effectiveness [12, 13]. We previously conducted a feasibility study to predict changes in BP from the start of anesthesia induction to surgical incision using a recurrent neural network (RNN) model and found that BP changes were predictable to some extent [14]. As a follow-up study, we became interested in whether BP was predictable in various special situations that may occur after the start of surgery. Among them, a model predicting BP fluctuations tailored to complex and special situations in lower abdominal laparoscopic surgery using da Vinci robots has yet to be developed. Therefore, a model based on deep learning (DL) that can predict BP fluctuations (especially BP increases) in these operations was developed to enable anesthesiologists to manage BP fluctuations and thereby reduce perioperative complications.

We empirically recognized that there were more cases of increased than decreased BP after the initiation of pneumoperitoneum and the Trendelenburg position during robotic low abdominal surgery. Hence, we hypothesized that it would be possible to use DL to predict whether BP would rise during abdominal pressure and position change. The purpose of this study was to develop a DL algorithm that could predict the occurrence of hypertension during robotic surgery 10 minutes after the start of pneumoperitoneum.

## Materials and methods

### Materials

**Fig 1:**
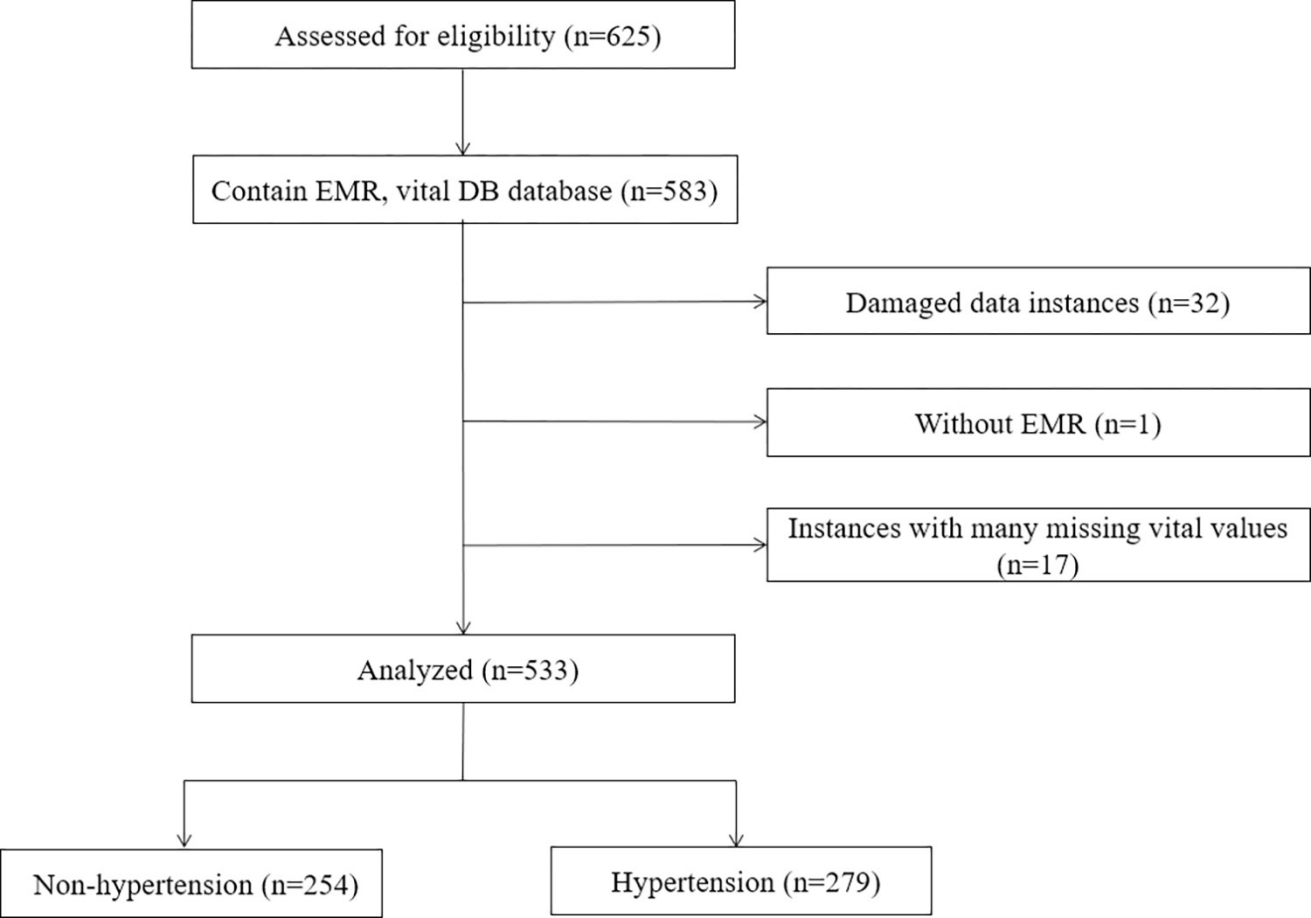
Flow diagram outlining data filtering strategy.

This study was approved by the Institutional Review Board of Soonchunhyang University Bucheon Hospital (approval No. 2021-05-026) and the requirement for informed consent was waived because all data were obtained by retrospective chart review. Databases that included adult patients (over 19 years old) treated with low abdominal da Vinci robot surgery (robotic-ovarian cystectomy, hysterectomy, myomectomy, prostatectomy, and salpingo-oophorectomy) at Soonchunhyang University Bucheon Hospital from October 1, 2018, to March 31, 2021, were selected. The data were collected from one da Vinci robot operating room. Each patient data set was a combination of attributes obtained from two different sources, the conventional electronic medical record (EMR) database and the operation data server (ODS). The EMR database provides the general attributes of a given patient, and the values are preprocessed by scaling and one-hot representation. The ODS maintains vital values using Vital recorder software [15], which works in real-time to gather data from the Bx50 (patient monitor) and the Datex–Ohmeda (anesthesia machine) monitoring machines. The total number of enrolled patients was 625. Data from patients with an American Society of Anesthesiologists (ASA) class of 4 or higher, or when the Vital recorder or EMR file was not fully recorded were excluded from the analysis. Finally, data from 533 patients were analyzed (Fig 1).

**Fig 2:**
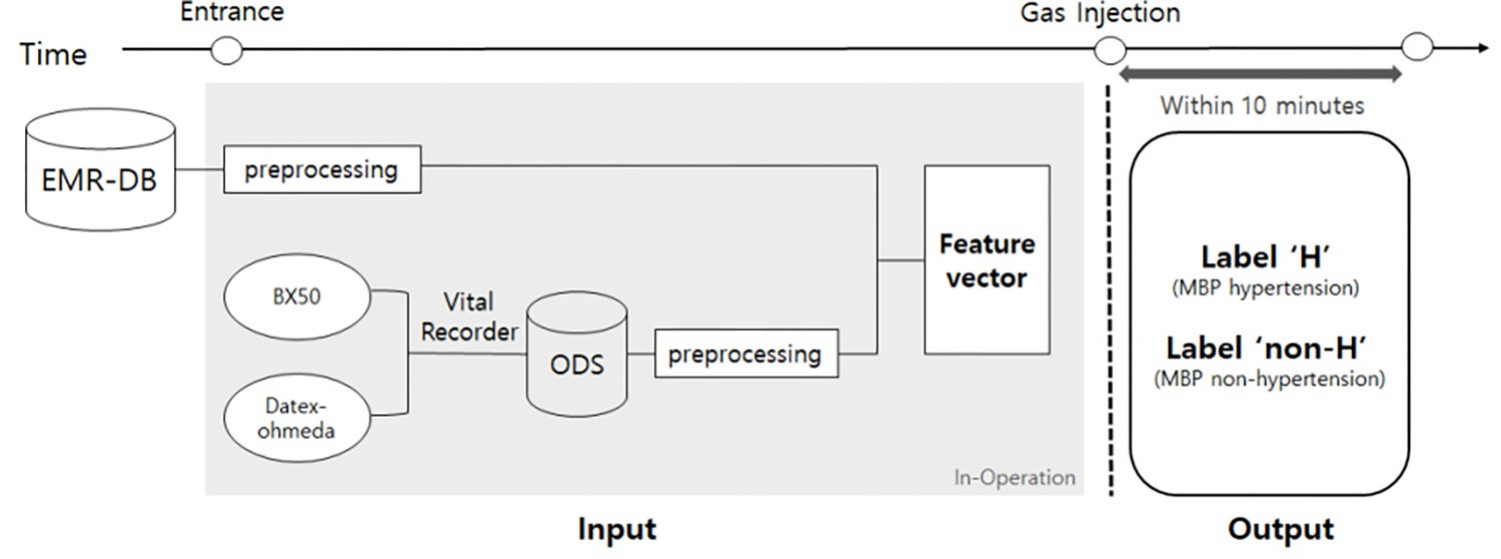
Overall process of the mean blood pressure hypertension prediction. EMR-DB, electronic medical record database; ODS, operation data server.

A feature vector from the attributes derived from the two sources (i.e., the EMR database and the ODS) was utilized for training a binary classification model for forecasting potential mean BP hypertension. Hypertension was defined as a 20% rise in the mean BP from baseline within 10 minutes after gas injection to the peritoneum. Two labels including “non-H” and “H” were used to indicate non-hypertension and hypertension, respectively. Fig 2 outlines the process flow. The purpose of this study was to demonstrate that our RNN model could predict non-H and H.

Based on the EMR database, several demographic information and patient status attributes were extracted including sex, age, weight, height, and ASA class. The categorical attributes such as sex and ASA class were converted into one-hot representations, while other numerical attributes (e.g., age, weight, and height) were maintained as real numbers, resulting in an 8-dimensional vector *Femr*. The attributes are listed in S1 Appendix. The 19 vital values were extracted from the ODS to obtain a real-numbered attribute matrix **F***vital* ∈ R^W×19^, where *W* is the size of the time window. The matrix **F***vital* had no missing values, but the vital values collected from different devices (or different ways) showed different sampling rates. For example, tidal volume and minute ventilation (MV) were collected every six seconds, whereas BP data were obtained every minute. Preprocessing was conducted as reported in a previous study [14], and thus, all vital values had the same sampling rate of 3 seconds, i.e., the size of time-window *W* was 60 using a vital value of 3 minutes. All numerical attributes in *Femr* and **F***vital* were normalized using a 0–1 scaling algorithm with only training data. S2 Appendix describes the per-label number of instances from a total of 533 instances.

### Methods

**Fig 3:**
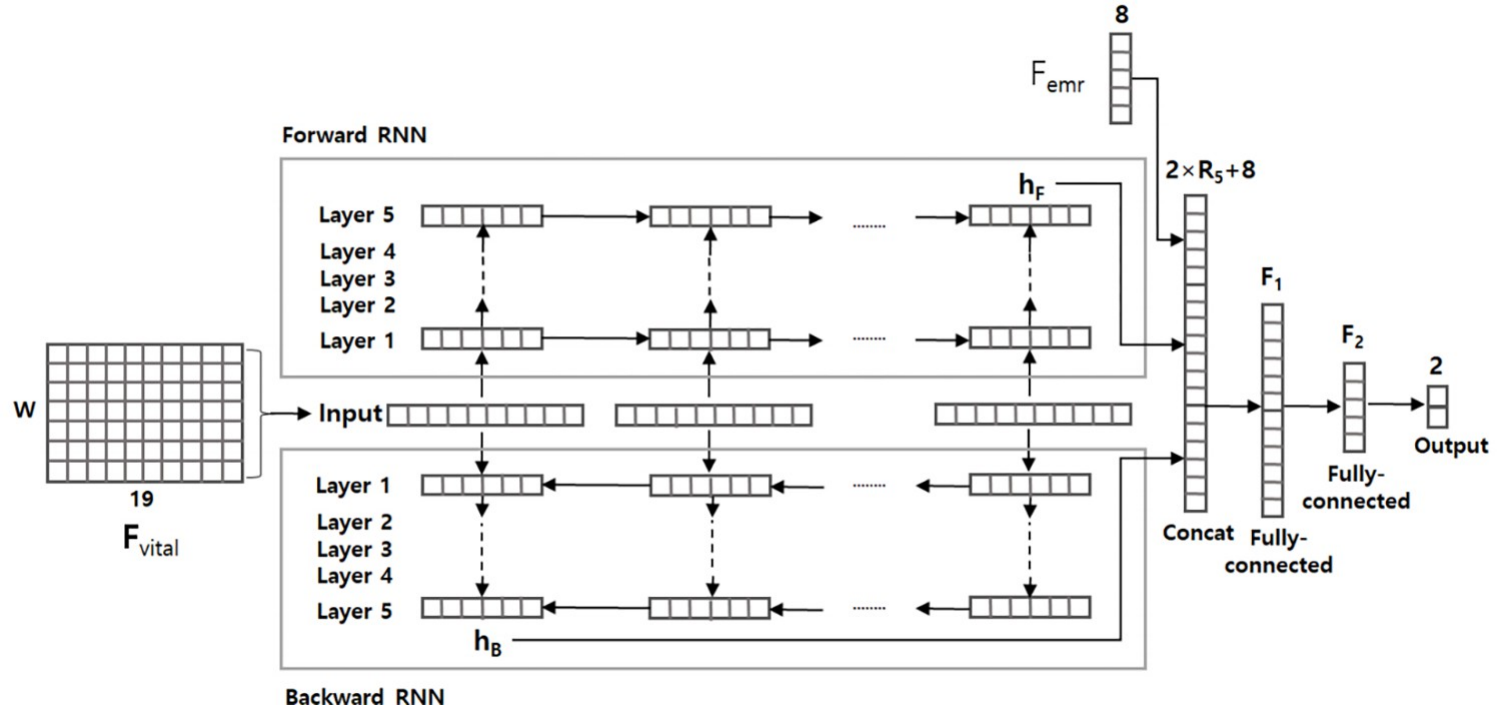
Recurrent neural network (RNN)-based model architecture.

An RNN-based model was designed using Python3 language with the PyTorch packages. The architecture is shown in Fig 3. The key architecture of the RNN-based model was the recurrent connections in hidden layers, and the output of the hidden layers, which conveyed the sequential patterns underlying the given data. The model used **F***vital* and *Femr* as input and generated a 2-dimensional output vector for prediction. The matrix **F***vital* represents a sequence of 19-dimensional vital values with the window size *W*. The sequence was fed into a stack of bidirectional gated recurrent unit [16] layers, resulting in two *R*_5_-dimensional real-numbered vectors, *h*_F_ and *h*_B_. The *h*_F_ vector was generated by the forward RNN and the *h*_B_ vector was derived from the backward RNN. These two vectors revealed the sequential patterns in the forward and backward directions, respectively. These vectors were concatenated with another input, the 8-dimensional real-numbered vector *Femr*, transforming into a 2 × *R*_*5*_ + 8-dimensional vector. The vector *Femr* consisted of independent features (e.g., age, sex, and ASA), and therefore, was not passed to the RNN layers but was concatenated with the vectors generated by the RNN layers. The concatenated vector was delivered to two consecutive fully connected (FC) layers followed by a 2-dimensional output layer. The two nodes of the output layer indicated hypertension and non-hypertension, respectively. The proposed RNN architecture was found via grid searching, such as by repeated experiments with training and validation datasets to identify the most promising number of RNN layers. The dimension of all RNN hidden layers (i.e., R1, R2, R3, R4, and R5) was set to 15. The time window size *W* = 10 suggests that the RNN model incorporated the vital sequences for 30 seconds as input. The dimensions of the two FC layers, F1 and F2, were 15 and 10, respectively. A cross-entropy loss function was used for parameter estimation.

### Statistical analysis of patient’s demographic data

Data analysis was conducted using SPSS software version 21.0 (SPSS Inc., Chicago IL, USA). The Kolmogorov-Smirnov test was performed to test the normality of the continuous variables. The Student’s *t*-test or Mann-Whitney *U* test was used depending upon the normality of the continuous variables. The chi-squared test or Fisher’s exact test was used for categorical variables. The Kruskal-Wallis test or oneway analysis of variance (ANOVA) along with post hoc analysis using Bonferroni’s method was used for multiple group comparisons. A *P*-value of less than 0.05 was considered significant.

There is no absolute sample size for training machine learning and deep learning models. The complexity of the model and training parameters are determined based on the amount and nature of the data. For that reason, it is often used to classify data into training, validation, and test datasets. Grid searching method is the most widely used method to obtain proof that model training has been performed properly for given data [17], and this method was also used in this study.

### Performance evaluation

The performance of the RNN model was evaluated using precision, recall, and F1 score. The evaluation was based on whether the model matched actual answers. The formula for each is as follows:

Precision = (True positive)/(True positive + False positive)

Recall = (True positive)/(True positive + False negative)

F1 Score = 2 * (Precision*Recall)/(Precision + Recall)

Macro average = Simple average of the values (F1, precision, recall) in each group.

Micro average = Weighted average of the values (F1, precision, recall). First, the values in each group were multiplied by the number of cases in each group, and the sum of these values was divided by the total number of cases.

## Results

The patient demographics in each of the non-H and H groups are listed in Tables 1 and 2. There were no differences in age, height, weight, body mass index (BMI), or comorbidities between the two groups, but the differences between the two groups were significant when surgery type and composition were considered. Therefore, the analysis of the differences in patient demographics based on surgery type revealed significant differences in age, height, weight, BMI, and comorbidities between the patient groups (S3 Appendix and Table 3).

**Table 1:** Patient demographics based on non-hypertension and hypertension group classification.

	All cases	Non-hypertension	Hypertension	*P*-value^a^
	(n = 533)	(n = 254, 47.7%)	(n = 279, 52.3%)	
Age (years)	48 (40–61)	46 (36–63)	49 (43–60)	0.044
Sex (male/female)	133/400	73/181	60/219	0.057
Height (cm)	161.11 ± 6.83	161.80 ± 7.34	160.47 ± 6.29	0.250
Weight (kg)	61.2 (54.6–69.8)	61.0 (54.9–70.9)	61.4 (54.1–61.4)	0.590
BMI (kg/m^2^)	23.7 (21.4–26.4)	23.4 (21.4–26.8)	24.0 (21.4–26.1)	0.653
Type of surgery				<0.001
Cystectomy (ovary)	86 (16.1)	55 (21.7)	31 (11.1)	
Hysterectomy	204 (38.3)	66 (26.0)	138 (49.5)	
Myomectomy	75 (14.1)	44 (17.3)	31 (11.1)	
Prostatectomy	129 (24.2)	70 (27.6)	59 (21.1)	
Salpingo-oophorectomy	39 (7.3)	19 (7.5)	20 (7.2)	
ASA classification				0.213
1	296 (55.5)	138 (54.3)	158 (56.6)	
2	207 (38.8)	97 (38.2)	110 (39.4)	
3	30 (5.6)	19 (7.5)	11 (3.9)	

The data are presented as the mean ± standard deviation, median (interquartile range), or n (%)

BMI, body mass index; ASA, American Society of Anesthesiologists

^a^Statistical significance was tested using the Mann-Whitney U test (age, weight, and BMI), *t*-test (height), or chi-squared test (type of surgery and ASA classification)

**Table 2:** Underlying comorbidities of patients based on non-hypertension and hypertension group classification.

Underlying diseases	All cases	Non-hypertension	Hypertension	*P*-value^a^
	(n = 533)	(n = 254)	(n = 279)	
Cardiovascular				
Hypertension	140 (26.3)	68 (26.8)	72 (25.8)	0.844
Atrial fibrillation	8 (1.5)	4 (1.6)	4 (1.4)	1.000
Coronary artery disease	3 (0.6)	3 (1.2)	0 (0.0)	0.108
Angina pectoris	4 (0.8)	3 (1.2)	1 (0.4)	0.352
Respiratory				
Asthma	22 (4.1)	8 (3.1)	14 (5.0)	0.384
COPD	3 (0.6)	1 (0.4)	2 (0.7)	1.000
Gastrointestinal				
Liver cirrhosis	4 (0.8)	4 (1.6)	0 (0.0)	0.051
Renal				
Chronic kidney injury	9 (1.7)	8 (3.1)	1 (0.4)	0.160
End-stage renal disease	2 (0.4)	1 (0.4)	1 (0.4)	1.000
Endocrine				
Diabetes mellitus	46 (8.6)	26 (10.2)	20 (7.2)	0.220
Thyroid disease	28 (5.3)	14 (5.5)	14 (5.0)	0.847
Neurologic				
Cerebrovascular disease	10 (1.9)	3 (1.2)	7 (2.5)	0.345

The data are presented as n (%)

COPD, chronic obstructive pulmonary disease

^a^Statistical significance between the groups were tested using the chi-squared test (hypertension, asthma, diabetes mellitus, and thyroid disease) or Fisher’s exact test (atrial fibrillation, coronary artery disease, angina pectoris, COPD, liver cirrhosis, chronic kidney injury, end-stage renal disease, and cerebrovascular disease)

**Table 3:** Underlying comorbidities of patients based on surgery type.

Underlying diseases	Cystectomy	Hysterectomy	Myomectomy	Prostatectomy	Salpingo-oophorectomy	*P*-value^a^
	(n = 86)	(n = 204)	(n = 75)	(n = 129)	(n = 39)	
Cardiovascular						
Hypertension	4 (4.7)	40 (19.6)	6 (8.0)	81 (62.8)	9 (23.1)	<0.001*
Atrial fibrillation	1 (1.2)	1 (0.5)	0 (0.0)	6 (4.7)	0 (0.0)	0.018*
Coronary artery disease	0 (0.0)	0 (0.0)	0 (0.0)	3 (2.3)	0 (0.0)	0.051
Angina pectoris	0 (0.0)	0 (0.0)	0 (0.0)	4 (3.1)	0 (0.0)	0.013*
Respiratory						
Asthma	2 (2.3)	10 (4.9)	4 (5.3)	5 (3.9)	1 (2.6)	0.817
COPD	0 (0.0)	0 (0.0)	0 (0.0)	3 (2.3)	0 (0.0)	0.051
Gastrointestinal						
Liver cirrhosis	1 (1.2)	1 (0.5)	0 (0.0)	2 (1.6)	0 (0.0)	0.671
Renal						
Chronic kidney injury	1 (1.2)	0 (0.0)	1 (1.3)	7 (5.4)	0 (0.0)	0.004*
End-stage renal disease	0 (0.0)	0 (0.0)	0 (0.0)	2 (1.6)	0 (0.0)	0.179
Endocrine						
Diabetes mellitus	1 (1.2)	16 (7.8)	2 (2.7)	27 (20.9)	0 (0.0)	<0.001*
Thyroid disease	2 (2.3)	16 (7.8)	2 (2.7)	3 (2.3)	5 (12.8)	0.018*
Neurologic						
Cerebrovascular disease	0 (0.0)	3 (1.5)	0 (0.0)	6 (4.7)	1 (2.6)	0.067

The data are presented as n (%)

COPD, chronic obstructive pulmonary disease

^a^Statistically significant differences between the groups were analyzed using the chi-squared test.

**Fig 4:**
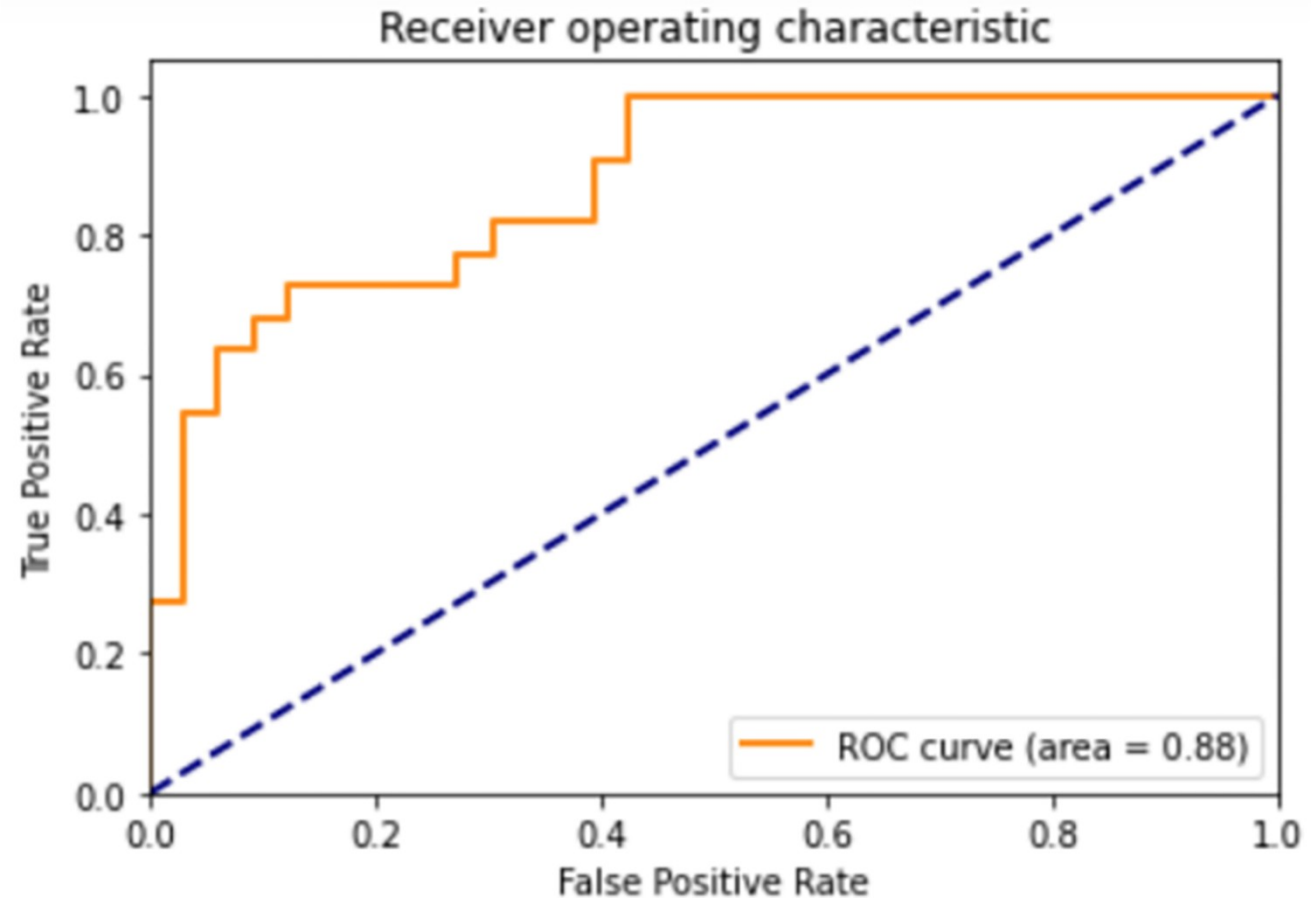
Receiver operating characteristic (ROC) curve of the proposed model with D9:1.

Using the total dataset D*total* (n = 533), three distinct datasets with different proportions of test data were generated. Five additional datasets were created using each of the five surgeries as test data. These eight derived datasets were generated from D*total* (S4 Appendix). The three datasets, D7:3, D8:2, and D9:1, were generated by random sampling but maintained the proportion of the different surgeries. The remaining datasets treated a particular type of surgery as test data. For example, D*sal* indicated that all instances of salpingo-oophorectomy surgery constituted the test data. Note that all datasets were the same size as the total dataset D*total*. Ten independent experiments were conducted with each dataset. All the reported performances (e.g., F1 score, precision, recall) represent the average values. The machine contained two central processing units of Intel(R) Xeon(R) Silver4214 at 2.20GHz and four graphics processing units of the NVIDIA Quadro RTX 5000. When the proposed model was trained, the optimal training recipe (e.g., number of epochs, drop-out probability, parameter initialization, learning rate, etc.) was found via a grid search with validation data, where the train:validation ratio was 9:1. Fig 4 shows the receiver operating characteristic (ROC) curve generated by a separated run with D9:1. Adam optimizer [18] with an initial learning rate of 0.001. The early-stopping algorithm was adopted with at least 50 epochs, and the mini-batch size was 32. A drop-out [19] with a keep probability of 0.1 in the RNN layers was adopted. Table 4 summarizes the overall performance. The macro-average F1 scores of all datasets were 72.33% and 68.18% for non-hypertension and hypertension, respectively. Based on these results, the prediction using the proposed model showed about 70% confidence. The elapsed time was about 3.472 milliseconds for generating 39 prediction outputs.

**Table 4:** Performance of the proposed model with different datasets.

Dataset	F1 (%)		Precision (%)		Recall (%)	
	Non-H	H	Non-H	H	Non-H	H
D_*sal*_^a^	71.87	70.60	68.78	74.61	75.26	67.00
D_*pro*_^b^	72.53	55.39	64.50	70.95	82.86	45.42
D_*myo*_^c^	70.72	66.78	78.55	60.50	64.32	74.52
D_*hys*_^d^	57.61	70.08	47.83	82.43	72.42	60.94
D_*cys*_^e^	75.16	71.34	93.33	58.54	62.91	91.29
D9:1^f^	82.54	74.52	81.76	76.91	83.33	72.27
D8:2^f^	73.75	68.09	73.83	68.91	73.67	67.29
D7:3^f^	74.49	68.66	71.55	72.75	77.67	65.00
Mac.Avg.	72.33	68.18	72.52	70.70	74.06	67.97
Mic.Avg.	70.22	67.50	68.46	72.15	74.31	65.40

Non-H, non-hypertension; H, hypertension

^a^D_*sal*_: a dataset in which all instances of robotic salpingo-oophorectomy surgery were used as test data.

^b^D_*pro*_: a dataset in which all instances of robotic prostatectomy surgery were used as test data.

^c^D_*myo*_: a dataset in which all instances of robotic myomectomy surgery were used as test data.

^d^D_*hys*_: a dataset in which all instances of robotic hysterectomy surgery were used as test data.

^e^D_*cys*_: a dataset in which all instances of robotic cystectomy surgery were used as test data.

^f^D7:3, D8:2, D9:1: datasets in which the train:validation ratios were 7:3, 8:2, and 9:1, respectively.

## Discussion

In this study, the task of predicting the mean BP hypertension of patients who underwent robotic laparoscopic surgery was addressed using an RNN-based model incorporating vital sequences and EMR data as input. The model used the vital values for 30 seconds to capture arbitrary sequential patterns and utilized the patient EMR data as supplementary features to predict potential hypertension within 10 minutes after pneumoperitoneum. The effectiveness of the model was verified by experimental results with our collected data. The model architecture and training recipe was found using a grid search, which yielded macro-average F1 scores of 68.18% to 72.33%. The gap between the macro and micro averages was attributed to the difference (e.g., the proportion of test data) between the datasets. The performance degraded according to decreases in the proportion of the training data. For example, the F1 score in the D9:1 dataset was substantially greater than that obtained with D7:3, which is reasonable because the model strength increases if the number of learning materials is high. The F1 scores with D*hys* were consistent with the observation. It had the worst result because it contained the smallest number of training data. Interestingly, among the five datasets with test data involving different types of surgery, the best F1 scores were achieved with D*cys* even though it was not the dataset with the highest number of training data. S3 Appendix and Table 3 show that the patients who underwent cystectomy were younger and fewer patients had hypertension. Vaso-reactivity is increased in patients with essential hypertension, and therefore, even a small change in a sympathetic agent induces large BP fluctuations, which complicate the prediction of BP fluctuations. Arterial stiffness increases with age, which may increase systolic BP and pulse pressure. This difference in basal conditions affected the predictive power of the D*cys* dataset. The results of two datasets, D*hys* and D*pro*, were contrasting, showing high F1 scores for ‘H’ in D*hys* but high F1 scores for ‘non-H’ in D*pro*. The contrast can be explained by the opposite label proportion in the datasets. As the D*hys* dataset had more ‘H’ than ‘non-H’ instances, the model tended to yield ‘H’ as a prediction output. Likewise, the D*pro* dataset had ‘non-H’ instances, and the model yielded more ‘non-H’ as a prediction output.

There are several widely-adopted ML models for medical applications including logistic regression, support vector machine [20], decision tree, random forest [21], naive Bayes, extreme gradient boosting [22], and artificial neural networks (ANN). Lee et al. [23] proposed an ANN model to predict postoperative in-hospital mortality, and achieved an area under the ROC curve of 0.91 (95% confidence interval: 0.88–0.93). Jeong et al. [24] compared a few ML models for predicting the potential postoperative complications of patients diagnosed with end-stage renal disease. Even though ML models are effective in performing several medical tasks, a major limitation relates to their need for human intervention, entailing feature engineering. The performance of ML models strongly depends upon feature definition, which is expensive to obtain satisfactory performance with each individual task [25, 26]. The DL technique is one of the solutions that address this limitation. It does not require significant human effort but automatically learns arbitrary features (or patterns) from the dataset. However, it is worth noting that the domain knowledge of the target task needs to be examined before designing DL models. For example, decisions regarding input data are based on domain knowledge. While there are several well-known types of DL models, RNNs are known to be effective in capturing sequential patterns [27]. The RNN model has been adopted in various medical applications such as intensive care unit mortality risk prediction [28], predicting diabetes mellitus [29], and predicting specific targeted clinical events [30]. These studies generally utilized sequential data (e.g., vital signs and time-stamped electronic health records) as input for the RNN model so that the model learned to predict the desired output (i.e., adverse outcomes). The RNN has also been successfully used to predict BP values and BP-related events (e.g., hypertension). Peng Su et al. [31] proposed an RNN architecture to predict future BP values a few days in advance. Thus, the RNN model could be utilized to predict whether hypertension would occur within 10 minutes after pneumoperitoneum in robot assisted-laparoscopic low abdominal surgery.

As the output of the proposed model was an indicator of potential mean BP hypertension within 10 minutes after pneumoperitoneum, the model should generate the prediction results as early as possible. It only took about 3.472 milliseconds to generate 39 prediction outputs, suggesting that it is possible to inform the physicians of potential hypertension immediately after gas injection during surgery using our model.

Despite the exquisite experimental results and overall F1 scores of around 70%, there were several limitations to this study. First, the performance, especially recall in the ‘H’ cases, needs improvement. For the ‘H’ cases in cystectomy surgery, the proposed model achieved a recall of 91.29%, in contrast to the ‘H’ cases in prostatectomy surgery. Based on demographic differences, the patients who underwent prostatectomy were older and had underlying diseases, which may also suggest the increased difficulty in predicting BP fluctuations in the elderly or those with a number of underlying diseases. This limitation can be addressed by better model architecture or an alternative training recipe. Second, further data are needed to improve the overall model performance. Especially, as shown in Table 4, the performance of five datasets (e.g., Dsal, Dpro, Dmyo, Dhys, and Dcys) containing the test data of a particular target surgery was relatively lower than that of the other datasets. The collection of additional data will facilitate the creation of a robust model for different types of surgery. Third, patient data from other instruments such as the bispectral index that monitor the depth of anesthesia or the syringe pumps injecting anesthesia-related drugs are often missed in the vital database due to connection problems, so they were not used as input variables in this RNN model. If additional cases are available and these data are also used as variables, the performance of the model will be improved. Fourth, the data were obtained from a single source (a surgery robot in the same operating room). A robust model requires data from different surgery rooms, medical centers, and hospitals. Fifth, Since the model in this study was not a real-time prediction model, there are restrictions on applying this to individual patients. However, we are conducting research to develop real-time perioperative BP prediction models based on models developed using these tasks [14]. I think it will be a great help to create an accurate real-time blood pressure prediction model in the future through data accumulated with these model tasks. Sixth, the potential confounding factors such as incision time and depth of anesthesia were not considered in this model. As a limitation of the retrospective study, it was difficult to precisely specify the incision time. In contrast, the initiation time of the pneumoperitoneum could be accurately determined through the record of changes in abdominal pressure levels. However, it seems that the incision time would not have a significant impact on the result. Because, in general, the trocar is inserted immediately at the rt of the incision and the pneumoperitoneum is started within 1–2 minutes. It was difficult to include depth of anesthesia into the analysis either. It was difficult to obtain uniform data, because the records of values for depth of anesthesia were omitted in many cases and various equipments (i.e., bispectral index, entropy, and sedline) were used for measuring the depth of anesthesia. There is a need for additional prospective studies.

In conclusion, the current RNN model could predict hypertension within 10 minutes after changing to the Trendelenburg position with CO_2_ pneumoperitoneum during various types of robotic surgery. Further studies are needed to increase the overall predictive power based on other vital features (i.e, dose of anesthetics, intra-abdominal pressure, and angle of Trendelenburg position) to improve model performance and provide reliable information to anesthesiologists.

## Supporting information

S1 Appendix Attributes of the two different sources. (DOCX)

S2 Appendix Per-label number of data instances. (DOCX)

S3 Appendix Patient demographics based on surgery type. (DOCX)

S4 Appendix Eight experimental datasets. (DOCX)

S5 Appendix Underlying dataset. (XLSX)
