# Supplementary material for: Prediction of blood pressure changes associated with abdominal pressure changes during robotic laparoscopic low abdominal surgery using deep learning
Source: PLoS One. 2022 Jun 6;17(6):e0269468. doi: 10.1371/journal.pone.0269468 (PMC9200233; doi:10.1371/journal.pone.0269468)
Supplement: S1 Appendix — (DOCX) [file pone.0269468.s001.docx]

**Attributes of the two different sources.**

| **Source** | **Name** | **Meaning** |
| --- | --- | --- |
| EMR-DB | Sex (male) | This is 1 if the patient is male, otherwise 0. |
|  | Sex (female) | This is 1 if the patient is female, otherwise 0. |
|  | ASA (1) | This is 1 if the ASA class is 1, otherwise 0. |
|  | ASA (2) | This is 1 if the ASA class is 2, otherwise 0. |
|  | ASA (3) | This is 1 if the ASA class is 3, otherwise 0. |
|  | Age | Patient age |
|  | Weight | Patient weight in kilograms (kg) |
|  | Height | Patient height in centimeters (cm) |
| ODS | HR | Heart rate (number of heartbeats per minute) |
|  | PLETH_SpO_2_ | Percutaneous oxygen saturation |
|  | ETCO_2_ | End-tidal carbon dioxide |
|  | CO_2_ | Carbon dioxide |
|  | AMB_PRES | Ambient pressure |
|  | NIBP_SBP | Non-invasive systolic blood pressure |
|  | NIBP_MBP | Non-invasive mean blood pressure |
|  | NIBP_DBP | Non-invasive diastolic blood pressure |
|  | NM_TOF_CNT | Neuromuscular transmission train-of-four count |
|  | PLETH | Plethysmography |
|  | RR_TOTAL | Total respiratory rate |
|  | TV | Tidal volume |
|  | MV | Minute ventilation |
|  | PIP | Peak inspiratory pressure |
|  | MAWP | Mean airway pressure |
|  | COMPLIANCE | Airway compliance |
|  | PEEP_TOTAL | Total positive end-expiratory pressure |
|  | FiO_2_ | Fraction of inspired oxygen |
|  | SPI | Surgical pleth index |

EMR-DB, electronic medical record database; ASA, American Society of Anesthesiologists; ODS, operation data server
