# Supplementary material for: Prediction of blood pressure changes associated with abdominal pressure changes during robotic laparoscopic low abdominal surgery using deep learning
Source: PLoS One. 2022 Jun 6;17(6):e0269468. doi: 10.1371/journal.pone.0269468 (PMC9200233; doi:10.1371/journal.pone.0269468)
Supplement: S2 Appendix — (DOCX) [file pone.0269468.s002.docx]

**Per-label number of data instances.**

| **Surgery** | **Non-hypertension** | **Hypertension** |
| --- | --- | --- |
| Cystectomy (ovary) | 55 | 31 |
| Hysterectomy | 66 | 138 |
| Myomectomy | 44 | 31 |
| Prostatectomy | 70 | 59 |
| Salpingo-oophorectomy | 19 | 20 |
| Total | 254 | 279 |
