# Supplementary material for: Prediction of blood pressure changes associated with abdominal pressure changes during robotic laparoscopic low abdominal surgery using deep learning
Source: PLoS One. 2022 Jun 6;17(6):e0269468. doi: 10.1371/journal.pone.0269468 (PMC9200233; doi:10.1371/journal.pone.0269468)
Supplement: S3 Appendix — (DOCX) [file pone.0269468.s003.docx]

**Patient demographics based on surgery type.**

|  | **Cystectomy**  **(n = 86)** | **Hysterectomy**  **(n = 204)** | **Myomectomy**  **(n = 75)** | **Prostatectomy**  **(n = 129)** | **Salpingo-oophorectomy**  **(n = 39)** | ***P*-value^a^** |
| --- | --- | --- | --- | --- | --- | --- |
| Age (years) | 33.30 ± 12.79^b^ | 49.91 ± 7.77^c^ | 36.45 ± 7.08^b^ | 65.89 ± 5.79^d^ | 51.64 ± 12.18^c^ | <0.001^*^ |
| Height (cm) | 160.92 ± 6.18^b^ | 158.39 ± 5.85^c^ | 159.92 ± 5.61^b^ | 167.01 ± 6.18^d^ | 158.54 ± 5.36^c^ | <0.001^*^ |
| Weight (kg) | 57.68 ± 8.49^b^ | 61.39 ± 10.94^b^ | 60.23 ± 11.81^b^ | 71.12 ± 9.99^c^ | 60.51 ± 7.86^b^ | <0.001^*^ |
| BMI | 22.33 ± 3.62^b^ | 24.48 ± 4.33^c^ | 23.55 ± 4.48^b,c^ | 25.47 ± 3.17^c^ | 24.07 ± 3.39^c^ | <0.001^*^ |

The data are presented as the mean ± standard deviation

BMI, body mass index

^a^Statistically significant differences between the groups was tested by the Kruskal-Wallis test (age, weight, and BMI) or one-way analysis of variance (height).

^b,c,d^The same letters indicate a non-significant difference based on post-hoc analysis (Bonferroni’s method).
