# Supplementary material for: Prediction of blood pressure changes associated with abdominal pressure changes during robotic laparoscopic low abdominal surgery using deep learning
Source: PLoS One. 2022 Jun 6;17(6):e0269468. doi: 10.1371/journal.pone.0269468 (PMC9200233; doi:10.1371/journal.pone.0269468)
Supplement: S4 Appendix — (DOCX) [file pone.0269468.s004.docx]

**Eight experimental datasets.**

| **Dataset** | **Train & Validation** | **Test** |
| --- | --- | --- |
| D7:3^a^ | 371 | 162 |
| D8:2^a^ | 425 | 108 |
| D9:1^a^ | 478 | 55 |
| D*_sal_*^b^ | 494 | 39 |
| D*_pro_*^c^ | 404 | 129 |
| D*_myo_*^d^ | 458 | 75 |
| D*_hys_*^e^ | 329 | 204 |
| D*_cys_*^f^ | 447 | 86 |

^a^D7:3, D8:2, D9:1: datasets in which the train:validation ratios were 7:3, 8:2, and 9:1, respectively.

^b^D*_sal_*: a dataset in which all instances of robotic salpingo-oophorectomy surgery were used as test data.

^c^D*_pro_*: a dataset in which all instances of robotic prostatectomy surgery were used as test data.

^d^D*_myo_*: a dataset in which all instances of robotic myomectomy surgery were used as test data.

^e^D*_hys_*: a dataset in which all instances of robotic hysterectomy surgery were used as test data.

^f^D*_cys_*: a dataset in which all instances of robotic cystectomy surgery were used as test data.
